# Smaller volumes in the lateral and basal nuclei of the amygdala in patients with panic disorder

**DOI:** 10.1371/journal.pone.0207163

**Published:** 2018-11-07

**Authors:** Takeshi Asami, Ryota Nakamura, Masao Takaishi, Haruhisa Yoshida, Asuka Yoshimi, Thomas J. Whitford, Yoshio Hirayasu

**Affiliations:** 1 Department of Psychiatry, Graduate School of Medicine, Yokohama City University, Yokohama, Japan; 2 School of Psychology, University of New South Wales, Sydney, New South Wales, Australia; 3 Heian Hospital, Okinawa, Japan; Hamamatsu University School of Medicine, JAPAN

## Abstract

The amygdala plays an important functional role in fear and anxiety. Abnormalities in the amygdala are believed to be involved in the neurobiological basis of panic disorder (PD). Previous structural neuroimaging studies have found global volumetric and morphological abnormalities in the amygdala in patients with PD. Very few studies, however, have explored for structural abnormalities in various amygdala sub-regions, which consist of various sub-nuclei, each with different functions. This study aimed to evaluate for volumetric abnormalities in the amygdala sub-nuclei, in order to provide a better understanding neurobiological basis of PD. Thirty-eight patients with PD and 38 matched healthy control (HC) participants underwent structural MRI scanning. The volume of the whole amygdala, as well as its consistent sub-nuclei, were calculated using FreeSurfer software. Relative volumes of these amygdala sub-regions were compared between the two groups. Results showed significantly smaller volumes in the right lateral and basal nuclei in the patients with PD compared with the HC. Lateral and basal nuclei are thought to play crucial role for processing sensory information related with anxiety and fear. Our results suggest that these particular amygdala sub-regions play a role in the development of PD symptoms.

## Introduction

Panic disorder (PD) is an anxiety disorder characterized by recurrent panic attacks. It has a lifetime prevalence of about 3%, and typically shows a chronic course. PD has been associated with both decreased social functioning and lower quality of life, both of which have been closely associated with symptom severity [1, 2]. Elucidating the neurobiological basis of PD is thus an important endeavor as it could provide a target for the development of more effective treatments.

Recent neuroimaging studies have provided evidence that patients with PD show functional and structural abnormalities in limbic regions, frontal regions, and brainstem regions (see reviews of [3] and [4]). The amygdala has been particularly heavily implicated, consistent with its established role in fear and anxiety. For example, a previous single photon emission computed tomography study reported higher levels of glucose uptake in the amygdala in patients with PD compared with healthy control (HC) participants [5]. A positron emission tomography study reported that presynaptic and postsynaptic monoamine neurotransmitter serotonin (5-HT) 1A receptor binding was reduced in the amygdala in untreated patients with PD compared to HC [6]. Moreover, recent functional magnetic resonance imaging (MRI) studies have demonstrated abnormalities in blood oxygenation level dependent (BOLD) activation in the amygdala in patients with PD relative to HC [7–10].

In terms of structural neuroimaging studies, most previous region-of-interest (ROI)-based analyses (which used manual tracing) have demonstrated either significant or trend level smaller volumes of the amygdala in patients with PD compared with HC [11–13]. A whole-brain voxel-based morphometry (VBM) analysis has also shown significantly smaller volumes in amygdala in patients with PD relative to HC [14]. There are, however, some whole brain VBM analyses that found no group differences in the volume of amygdala [15, 16]. In light of this ambiguity, we thought it important to provide more evidence regarding the presence (or absence) of structural abnormalities in the amygdala in patients PD using the most recent technology.

The amygdala consists of several nuclei, including the lateral nucleus, basal nucleus, and central nucleus. Each of these nuclei are connected with various other brain regions, as well as other nuclei in amygdala. There is evidence to suggest that these nuclei each have discrete functions [17]. According to the dominant neuroanatomical hypothesis of PD [18], the activity of the amygdala is regulated by the medial prefrontal cortex (PFC), which includes the anterior cingulate cortex, insula, and thalamus. Signals from the amygdala (modulated by the PFC) are send to target brain regions including the hypothalamus and brainstem, and these signals precipitate the symptoms of PD. According to this hypothesis, the amygdala—particularly the lateral nucleus, basal nucleus, and central nucleus—plays a crucial role in the genesis of PD symptoms.

The present study aimed to evaluate whether structural abnormalities in amygdala, which have previously been observed at a global level, were also observable at a finer-grained level, in the various amygdala sub-nuclei. A previous study, from our laboratory, has detected a smaller volume in the corticomedial nucleus in patients with PD compared with HC, using an ROI-defined VBM analysis [13]. A previous morphometric (i.e., shape-based) analysis has also found minute structural abnormalities in the amygdala in patients with PD. Specifically, the PD patients demonstrated inward deformation in laterobasal and centromedial nuclei in the amygdala compared with HC [19]. However, to the best of our knowledge, these are the only two studies which have investigated for minute structural abnormalities in amygdala; there are no previous studies which have calculated the volume of the amygdala sub-nuclei in patients with PD.

In the present study, we calculated the volume of each nucleus of amygdala using state-of-the-art analysis techniques, and conducted group comparisons between patients with PD and HC. We speculated that smaller volumes would be confirmed in lateral nucleus, basal nucleus, and central nucleus of the amygdala in PD patients relative to HC.

## Material and methods

### Subjects

Thirty-eight patients with PD (25 female and 13 male) and 38 HC (25 female and 13 male) participated in this study. These participants were the same as those described in our previous report [20], where demographic information is provided. Briefly, participants’ mean age was (mean ± standard deviation, [range]) 39.7 ± 9.9 [19–56] years old in the patients with PD and 39.0 ± 11.1 [22–58] years old in the HC. Participant’s/parental socioeconomic status (SES) [21] score was 2.7 ± 0.9 / 2.8 ± 0.8 in the patients, and 2.5 ± 0.8 / 2.4 ± 0.9 in the HC. There were no significant differences between the two groups in age, gender ratio or parental SES. In the PD group, patients’ mean duration of illness was 4.7 ± 5.8 years, their mean score on the Panic Disorder Severity Scale (PDSS) was 12.3 ± 5.5, and their mean score on the Global Assessment of Functioning (GAF) was 61.0 ± 9.3. Patients were recruited from inpatient and outpatient units of Yokohama City University Hospital, and all the patients were currently taking antidepressant and/or benzodiazepine medications [20]. HC were recruited from the community and hospital staff. This study was approved by the Medical Research Ethics Committee of Yokohama City University. After providing a complete description of the study, we obtained written informed consent from all participants.

### Image processing and volume comparison

MR images were acquired with a 1.5-T Magnetom Symphony system (Siemens Medical System, Erlangen, Germany) at Yokohama City University Hospital. A series of 128 contiguous, sagittal T1-weighted slices were acquired [20, 22]. A series of 128 contiguous T1-weighted slices in sagittal images was acquired with a Turbo FLASH sequence with the following parameters: echo time = 3.93 ms, repetition time = 1960 ms, inversion time = 1100 ms, flip angle = 15, field of view = 24 cm, matrix = 256x256x128, and voxel dimensions = 0.9375x0.9375x1.5 mm.

**Fig 1:**
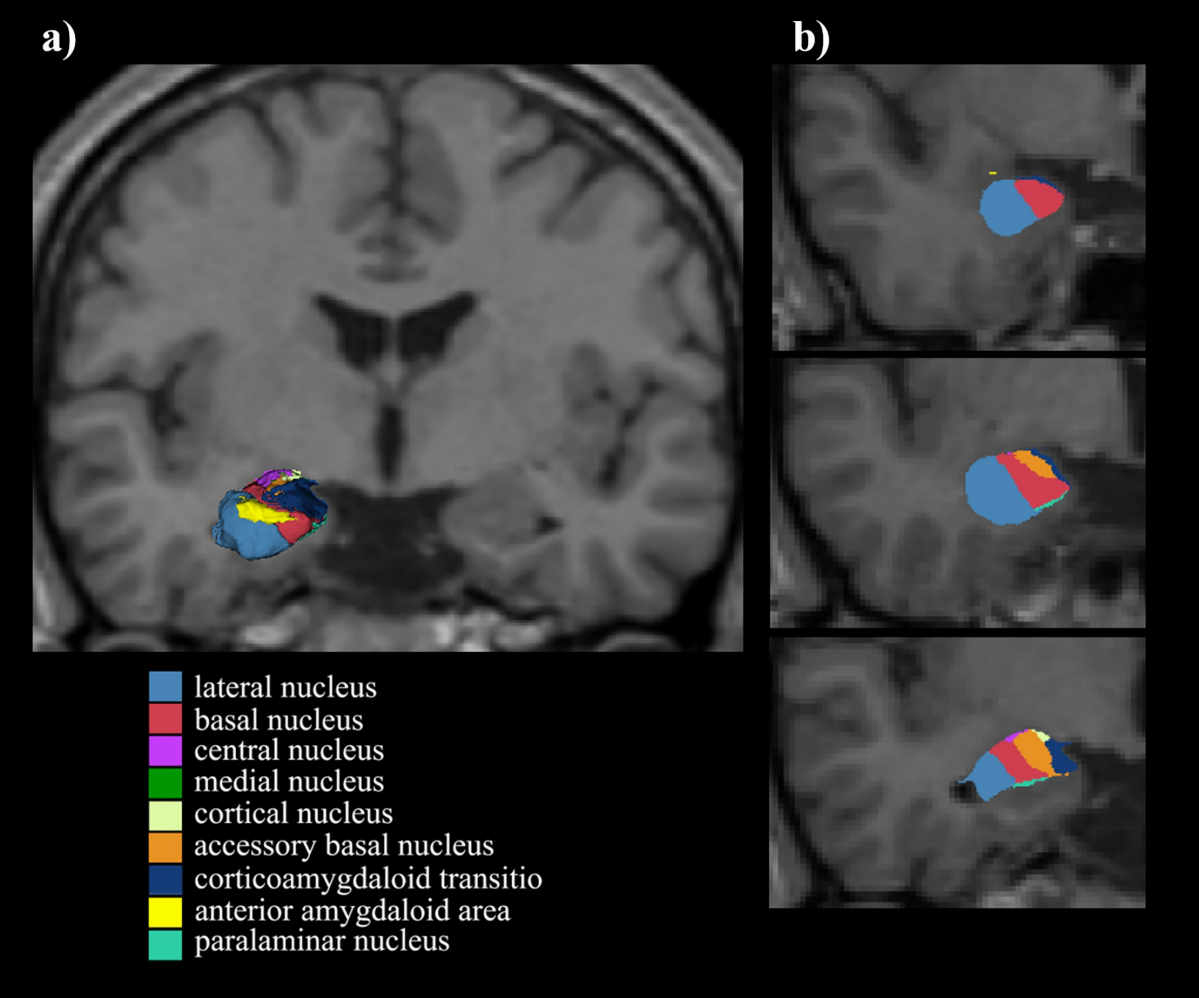
Nuclei of the amygdala. a) 3D image was constructed using 3D-Slicer. b) Coronal images of the nuclei in the right amygdala.

Image analysis was employed using the FreeSurfer software (ElCapitan-development version; http://surfer.nmr.mgh.harvard.edu/). The technical details of the procedures have been described elsewhere [23]. In short, the image procedure included motion correction, intensity normalization, skull stripping, segmentation of white matter, tessellation of the grey/white matter boundary, automated topology correction, and surface deformation. The volume of whole amygdala, as well as the volumes of its nine constituent nuclei were calculated for each hemisphere. The nine nuclei were: lateral nucleus, basal nucleus, central nucleus, medial nucleus, cortical nucleus, accessory basal nucleus, corticoamygdaloid transition, anterior amygdaloid area, and paralaminar nucleus [24] (**Fig 1**). Intracranial content volumes were obtained via a MATLAB function, and relative volumes of these regions were calculated.

To assess group differences between the patients with PD and HC in the volume of the whole amygdala and each of its constituent nuclei, univariate covariance analyses controlling for age and sex were employed. Significance threshold was set at P < .025 for volume comparison of the whole amygdala. For the comparison of each nucleus, P < .008 (i.e., .05/ 6 regions) was adopted for the lateral, basal, and central nuclei because of the hypothesis-driven nature of these analyses, and P < .0028 (i.e., .05/ 18 regions) was used for the other nuclei. For any region in which volumetric differences were observed, Pearson correlation analyses were conducted between the volume of the structure(s) in question and patients’ scores on the PDSS and GAF. A critical p-value of p < .05 was used for the correlational analysis.

## Results

The patients with PD showed a significantly smaller volume in the right whole amygdala compared with the HC. In the sub-nuclei analysis, significant smaller volumes in the right lateral nucleus and the basal nucleus were demonstrated in the PD patients compared to the HC (**Table 1**). There were no group differences in the volume of the central nucleus, or any other nuclei. Correlation analyses showed no significant associations between volumes of the right lateral and basal nucleus and scores of the PDSS or GAF in the patients with PD.

**Table 1:** Relative volume of whole amygdala and each amygdala nucleus.

	PD (n = 38)		HC (n = 38)			
region	mean ^a^	SD	mean ^a^	SD	F	p
rt. whole amygdala	4.21	0.28	4.40	0.25	9.44	.003*
rt. lateral nucleus	1.58	0.11	1.66	0.11	8.86	.0040**
rt. basal nucleus	1.07	0.09	1.13	0.09	10.50	.0018**
rt. accessory basal nucleus	0.66	0.06	0.68	0.06	3.07	.084
rt. anterior amygdaloid area	0.15	0.02	0.15	0.02	.70	.406
rt. central nucleus	0.11	0.02	0.12	0.018	.08	.780
rt. medial nucleus	0.055	0.013	0.056	0.013	.08	.777
rt. cortical nucleus	0.068	0.008	0.070	0.009	1.72	.194
rt. corticoamygdaloid transitio	0.41	0.04	0.43	0.04	3.23	.076
rt. paralaminar nucleus	0.12	0.01	0.12	0.01	.08	.784
lt. whole amygdala	4.12	0.35	4.22	0.25	1.96	.165
lt. lateral nucleus	1.63	0.15	1.68	0.14	2.19	.143
lt. basal nucleus	1.02	0.10	1.04	0.06	1.13	.290
lt. accessory basal nucleus	0.61	0.06	0.62	0.05	1.41	.239
lt. anterior amygdaloid area	0.14	0.02	0.13	0.01	1.41	.240
lt. central nucleus	0.11	0.02	0.11	0.02	.04	.894
lt. medial nucleus	0.053	0.015	0.051	0.012	.37	.543
lt. cortical nucleus	0.062	0.008	0.063	0.008	.56	.458
lt. corticoamygdaloid transitio	0.39	0.03	0.40	0.04	4.23	.043
lt. paralaminar nucleus	0.11	0.01	0.12	0.01	3.43	.068

Abbreviations: PD, panic disorder; HC, healthy control subject; rt, right; lt, left

^a^ relative volume [= (absolute volume / intracranial content volume) x 100] (%) x 1000

* P < .025

** P < .008

## Discussion

To the best of our knowledge, this is the first study to evaluate the volume of the amygdala sub-nuclei in patients with PD. The results demonstrated that the PD patients exhibited significantly smaller volumes in the right lateral nucleus and basal nucleus of the amygdala, compared with the matched HC participants.

In the current study, we first confirmed that the patients with PD exhibited significantly smaller volumes of the right (whole) amygdala, relative to the HC. This result is in line with previous volumetric studies which have used manual tracing methods and observed smaller global volumes of the amygdala in PD patients [11–13], as well as one automated study which used a whole brain VBM analysis [14]. Given the consistency of this finding, it is possible that volumetric deficits may represent the structural basis of the functional abnormalities in the amygdala that have consistently been reported in PD patients in the neuroimaging literature [5–10].

Our sub-regional analyses revealed significantly smaller volumes in the right lateral nucleus and the basal nucleus of the amygdala in the patients with PD compared with the HC. This result is in line with the previous finding that patients with PD showed inward shape deformation in the laterobasal nuclei of the right amygdala compared with HC [19]. Our result is also consistent with previous functional imaging studies which have reported activations in the basolateral amygdala in response to fear-related stimuli in the healthy volunteers [25, 26].

Anatomically, the lateral nucleus in amygdala is believed to share connections with the sensory thalamus, medial PFC, anterior cingulate cortex, and superior temporal gyrus, and to receive sensory information from these brain regions [17]. According to Whalen and Phelps (2009), this sensory information is then transmitted to both the basal nucleus and intercalated nucleus in the amygdala. The basal nucleus also receives input from medial PFC and orbitofrontal cortex, in addition to the lateral nucleus, and is thus believed to play an important role in regulating cognition and emotion. Next, the sensory information is transferred to the central nucleus. The central nucleus has anatomical projections to brainstem regions and the hypothalamus. The theory posits that anxiety and fear-related responses are provoked by the information conveyed to these brain regions from the amygdala nuclei. In addition to these anatomical networks, the lateral and basal nuclei are believed to be connected with other brain regions related with anxiety and fear responses; for example, these nuclei have reciprocal connections with the insula, which is involved in the regulation of autonomic functions. The neural pathway from the basal nucleus to the ventral striatum is also believed to be related with regulation of fear-related behaviors [17].

As hypothesized by Whalen and Phelps (2009) [17], in light of the anatomical connections between the lateral, basal, and central nuclei, and other brain regions, sensory information related with anxiety and fear may be processed as follows. Sensory information, provoked by environmental stimuli, is first conveyed to the lateral nucleus of the amygdala, where an evaluation occurs as to whether the environmental stimuli are already known (or not), and whether the stimuli represent a threat to life (or not). Once a decision has been made that the stimuli do represent a threat to life, this information is conveyed to various brain regions, including the brainstem, via the central nucleus, where a general response is provoked. However, the general responses are only provoked in the situation in which the environmental stimuli are recognized, and this judgement is made in the basal nucleus. The basal nucleus receives input not only from the lateral nucleus of the amygdala, but also from the orbitofrontal cortex. The basal nucleus works as coincidence detector. If a potential crisis is detected, and the individual is judged as being in a dangerous situation, the basal nucleus activates the central nucleus. If, however, the individual is judged as being a safe situation, the signal of fear is caught by the filter of basal nucleus and is not conveyed to central nucleus.

The current study demonstrated smaller volumes in the lateral and basal nuclei of the amygdala in patients with PD. These structural deficits may represent the neurobiological basis of the PD symptoms; specifically, as speculated above, the lateral nucleus may misjudge sensory information to include a threat to the participant’s life, while the basal nucleus may midjudge the dangerousness of the situation. This misprocessed information may then be conveyed to the brainstem and hypothalamus, via the central nucleus, resulting in the occurrence of the PD symptoms.

In the correlation analyses, volumes of the right lateral and basal nuclei showed no relationships with scores of the PDSS. The PDSS evaluated patient’s symptom severity and degree of social and occupational dysfunction, and thus it might be predicted that the PDSS would be associated with brain regions which were related to occurrence of PD symptoms directly (e.g., brain stem regions), or regions associated with higher cognitive functioning, such as the prefrontal cortex. Indeed, previous studies from our laboratory have shown relationships between scores of the PDSS and volumes of the midbrain [27] and cortical thickness in the middle frontal cortex [20].

This study has some limitations worth noting. Firstly, the current study showed significant smaller volumes of the right lateral and basal nuclei in the patients with PD in the hypothesis driven analysis with cut-off P < .008. However, volume difference in the right lateral nucleus was not significant in the stricter threshold of the Bonferroni correction with P < .0028, necessitating further confirmation and limiting the interpretation of the findings. Second, all patients were receiving antidepressant and/or benzodiazepine medication at the time of scanning. Previous studies about PD have reported gender differences in both the epidemiology and symptom profile [1], as well as gender-related differences in the level of volumetric abnormalities of amygdala [14]. Thus, studies with drug naïve patients in a larger patient sample would be worthwhile in the future. Finally, this analysis was conducted using relatively low-resolution images (1.5T-MRI data); and it would be worthwhile confirm our results using higher resolution data (e.g., 3T-MRI data) in the future study.

## Conclusion

In conclusion, our volumetric analysis of the sub-nuclei of the amygdala detected significantly smaller volumes in the lateral and basal nuclei in patients with PD. These regions may play an important role in the genesis of PD symptoms.
